# Analyses of Evolutionary Characteristics of the Hemagglutinin-Esterase Gene of Influenza C Virus during a Period of 68 Years Reveals Evolutionary Patterns Different from Influenza A and B Viruses

**DOI:** 10.3390/v8120321

**Published:** 2016-11-26

**Authors:** Yuki Furuse, Yoko Matsuzaki, Hidekazu Nishimura, Hitoshi Oshitani

**Affiliations:** 1 Department of Virology, Tohoku University Graduate School of Medicine, Sendai 9808575, Japan; oshitanih@med.tohoku.ac.jp; 2 Department of Infectious Diseases, Yamagata University Faculty of Medicine, Yamagata 9909585, Japan; matuzaki@med.id.yamagata-u.ac.jp; 3 Virus Research Center, Clinical Research Division, Sendai Medical Center, Sendai 9838520, Japan; hide_nishimura1955@yahoo.co.jp

**Keywords:** influenza C virus, evolution, phylogenetics

## Abstract

Infections with the influenza C virus causing respiratory symptoms are common, particularly among children. Since isolation and detection of the virus are rarely performed, compared with influenza A and B viruses, the small number of available sequences of the virus makes it difficult to analyze its evolutionary dynamics. Recently, we reported the full genome sequence of 102 strains of the virus. Here, we exploited the data to elucidate the evolutionary characteristics and phylodynamics of the virus compared with influenza A and B viruses. Along with our data, we obtained public sequence data of the hemagglutinin-esterase gene of the virus; the dataset consists of 218 unique sequences of the virus collected from 14 countries between 1947 and 2014. Informatics analyses revealed that (1) multiple lineages have been circulating globally; (2) there have been weak and infrequent selective bottlenecks; (3) the evolutionary rate is low because of weak positive selection and a low capability to induce mutations; and (4) there is no significant positive selection although a few mutations affecting its antigenicity have been induced. The unique evolutionary dynamics of the influenza C virus must be shaped by multiple factors, including virological, immunological, and epidemiological characteristics.

## 1. Introduction

The influenza C virus is predominantly found in humans, and infection in humans can cause respiratory and febrile symptoms that are similar to those caused by influenza A and B viruses. Influenza C virus infections are generally mild and self-limited but can also cause more severe lower respiratory tract illness, such as bronchitis and pneumonia [1,2]. The infection is considered common based on high seroprevalence [3,4,5]. The influenza C virus has also been found in pigs, and there are some reports about interspecies transmission of the virus between humans and pigs [6,7]. However, pigs are not considered to play a significant role in the transmission cycle of the viruses in humans [7,8].

The influenza C virus is a member of the *Orthomyxoviridae* family, enveloped and segmented negative-sense RNA virus with seven segments. The fourth segment of the viral genome encodes hemagglutinin-esterase (HE) glycoprotein [9], which determines the major antigenicity of the virus and has a variety of functions in the viral replication cycle. At least nine antigenic sites (A-1 to A-5 and B-1 to B-4) of the HE glycoproteins are proposed; of them, amino acid positions responsible for four antigenic sites have been identified [10,11,12]. In addition, glycosylation of the proteins also affects the virus’ antigenicity [10]. The influenza C virus utilizes the HE glycoprotein as an attachment protein to a cellular receptor of the virus, 9-*O*-acetyl-*N*-acetylneuraminic acid [13,14]. The HE glycoprotein also catalyzes fusion of the viral envelope with endocytic vesicles [15]. Proteolytic cleavage of the protein into two subunits, HE1 and HE2, is an essential prerequisite for the membrane fusion activity [9]. In addition, the HE glycoprotein has esterase activity that functions as the receptor-destroying enzyme to release the progeny of viral particles from infected cells [14].

The influenza C virus was first isolated in 1947 [16]. Since then, it has been reported that antigenically and genetically-distinct lineages of the virus are co-circulating [17,18]. In the influenza A virus, there are two subtypes currently circulating among human populations; A/H1N1 and A/H3N2. In contrast to the influenza C virus, antigenically and genetically-similar strains of seasonal influenza A virus are circulating worldwide at a given time; and new antigenic variants, which are descendants of formerly circulating viruses, replace the previous viruses and become a predominant strain [19,20,21]. The influenza B virus does not have subtypes, but currently circulating influenza B viruses are divided into two phylogenetically and antigenically distinct lineages: B/Victoria/2/87-like (B/Victoria) lineage and B/Yamagata/16/88-like (B/Yamagata) lineage [22,23].

The seasonal influenza A virus accumulates mutations in the hemagglutinin (HA) gene, which encodes a major surface antigenic protein of the virus [21,24]. The virus causes annual epidemics by continuous antigenic change (antigenic drift), which allows viruses to evade herd immunity [25]. Therefore, positive selection that results in cumulative mutations in antigenic sites of the HA gene through evolutionary history has been observed [26,27,28,29]. The HA protein of the influenza A virus can also gain or lose glycosylation sites, which can alter the antigenicity of the virus [30,31]. There is a selection pressure on antigenic sites in the HA gene of influenza B viruses as well, although it is considered weaker than that of the influenza A viruses [32,33]. Buonagurio et al. and Muraki et al. reported little or no accumulation of mutations in the HE gene of the influenza C virus in the 1980s and 1990s, respectively; although they analyzed limited numbers of sequences available then (<20 strains) [34,35].

Differences in ecological, epidemiological, and evolutionary characteristics between influenza A, B, and C viruses are of great interest. Apart from antigenic analyses using viral isolates and sera or monoclonal antibodies against the viruses [17,36], phylogenetic analyses using genetic sequence data of the HE gene could provide important information about the evolution of the influenza C virus. However, because isolation of the influenza C virus using cell cultures, such as MDCK and LLC-MK_2_ cells requires technical proficiency and intensive observation of inoculated cells, isolation of the virus is rarely performed. This is one of the reasons why sequence data of the virus are still limited.

We recently reported the analysis of the full genome sequence of 102 strains of the influenza C virus and unveiled a history of frequent reassortment of the virus [18]. Reports and sequence data of the virus from many parts of the world have also been increasing [37,38]. In this study, we exploited sequence data available to date, many of which are from our recent study, with phylogenetic techniques to see the evolutionary pathway of the influenza C virus over a period of 68 years. We also compared the evolution of influenza C viruses with those of influenza A and B viruses.

## 2. Materials and Methods

### 2.1. Sequence Data

Nucleotide sequences of the HE gene (nucleotide position from 64 to 1989 corresponding to the complete coding region excluding the signal peptide) of 102 influenza C viruses were analyzed as previously described [18]. In addition to our data, all available full-length nucleotide sequence data of the HE gene of the virus detected from humans were obtained from the Influenza Virus Resource [39] and GISAID [40]. Sequence data of the HA gene of human influenza A and B viruses were also obtained from the Influenza Virus Resource database; we randomly selected a maximum of 20 strains per year for each dataset: A/H1N1, A/H3N2, and B. Sequence data used are available in Supplementary Materials (Data S1–S4). Sequence data of C/Yamagata/10/81 (GenBank accession number: M11641) were excluded from phylodynamics analyses described below because the strain is suspected of cross-contamination with C/AnnArbor/50 [34]. For evolutionary analyses of the influenza A/H1N1 virus, sequence data collected between 1977 and 2008, and data collected in 2009 or after, were analyzed separately because the A/H1N1 virus which circulated before and after the pandemic in 2009 must have undertaken a different evolutionary pathway [29,41,42,43].

### 2.2. Phylogenetic Tree

The evolutionary history was inferred by using PhyML 3.0 with the maximum likelihood method based on the general time reversible model [44]. Gamma distribution was used to model the evolutionary rate differences between sites; the rate variation model allowed for some sites to be evolutionarily invariable. A fast approximate likelihood ratio test (aLRT) was calculated for branch supports [45]. The tree is drawn to scale, with branch lengths measured in the number of substitutions per site.

### 2.3. Phylodynamics

Time-scaled phylogenies were inferred using a relaxed molecular clock model in a Bayesian Markov chain Monte Carlo (MCMC) framework with the BEAST program version 1.8.2 that incorporates virus sampling dates (year) to concurrently estimate phylogenetic trees, rates of nucleotide substitution, and the dynamics of population genetic diversity using a coalescent-based approach [46]. We performed at least 20 million generations sampled every 1000 runs. After the appropriate removal of burn-in (10% of samples), a summary maximum clade credibility (MCC) tree was inferred. The MCC trees were also used to estimate the time to the most recent common ancestor (tMRCA) among contemporaneous (same year) and posterior (at least three years) strains [47,48]. tMRCA of influenza A/H1N1 (before 2009) and influenza A/H1N1pdm (in or after 2009) was calculated separately because the A/H1N1pdm virus is not directly descended from the previously-circulating A/H1N1 seasonal virus [43]. Evolutionary rate of influenza A/H1N1pdm virus were not analyzed in this study because of insufficient duration of circulation of the virus (ca. six years). The past population dynamics of each lineage were compared using a Bayesian skyride analysis in BEAST, which utilizes a Gaussian Markov random field (GMRF) smoothing to estimate the changes in relative genetic diversity in successive coalescent intervals [49].

### 2.4. Hemagglutination Inhibition Test

The hemagglutination inhibition (HI) test was done as previously described [50]. Briefly, viruses were isolated from throat swabs by inoculating them into the amniotic cavity of nine-day-old embryonated hen’s eggs. Fifty microliters of 16 hemagglutinating units (HAU) of virus suspension per ml was added to each well containing 50 μL of two fold-diluted monoclonal antibodies [10,12]. After incubation for 30 min at room temperature, 100 μL of 0.5% chicken erythrocytes was added to all wells, and plates were stored for 60 min at 4 °C. The HI titer was expressed as the reciprocal of the highest antibody dilution which completely inhibited hemagglutination.

### 2.5. Selection Pressure

Relative numbers of non-synonymous and synonymous substitutions (dN and dS, respectively) through evolutionary history were analyzed to see selective pressure by HyPhy version 2.2.4 for the entire gene (global model) and for each codon (single-likelihood ancestor counting(SLAC) method) [51,52]. Selection pressure of influenza A/H1N1pdm virus were not analyzed in this study because of insufficient duration of circulation of the virus (ca. six years). Sites where dN–dS showed positive and negative values were inferred as positively- and negatively-selected, respectively. A *p* value less than 0.05 was considered statistically significant.

## 3. Results

### 3.1. Phylogenetic Tree and Population Dynamics of Influenza C Virus

**Figure 1:**
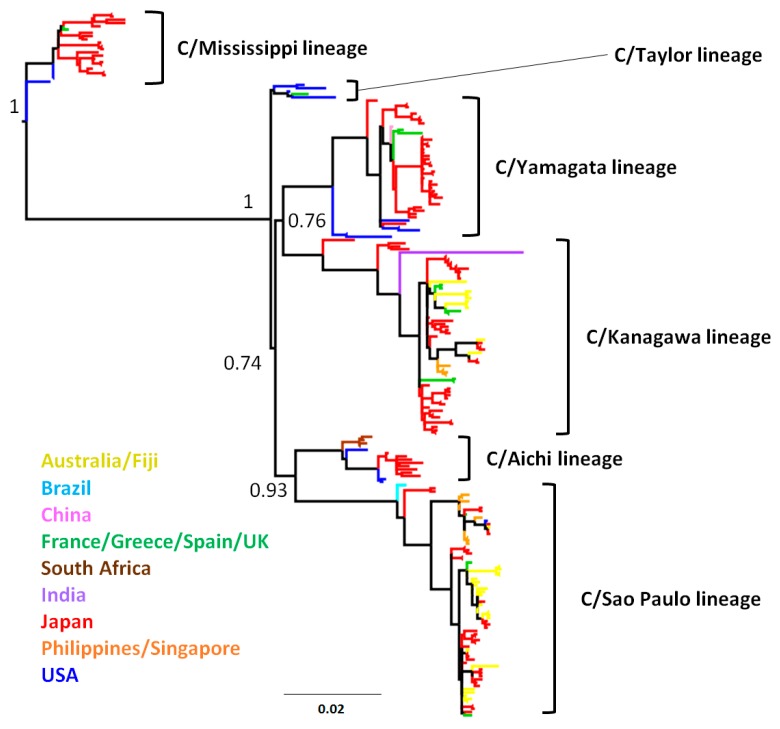
Phylogenetic tree for the hemagglutinin-esterase (HE) gene. The evolutionary history was inferred using the PhyML method for the HE gene (1917 positions) of influenza C viruses. Genetic lineages were defined by our previous study [18]. Approximate likelihood ratio test (aLRT) branch supports at the node to define each lineage are shown. Branches are colored by country.

Of all sequence data of the full length of the HE gene (excluding a region encoding signal peptide) of the influenza C virus collected from 14 countries between 1947 and 2014, we identified 218 unique sequences. Of the 218 strains, 137 were from Japan, 32 were from Australia, and 15 were from the USA (Supplementary Table S1). These 218 strains were classified into six genetically distinct lineages (Figure 1); they correspond to antigenic groups, as previously reported [17,18]. Although most strains were collected in Japan, strains from other countries were also scattered in the phylogenetic tree without any particular geographic clustering. For example, viruses in all lineages, except the C/Aichi lineage, were found in Europe. This suggests that there is no “indigenous” lineage, and multiple lineages are circulating globally.

**Figure 2:**
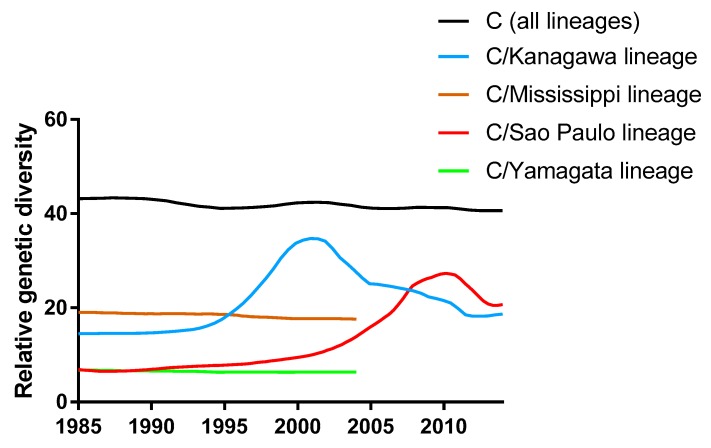
Population dynamics of genetic diversity of each lineage of the influenza C virus. The relative genetic diversity of each lineage of the influenza C virus using the Gaussian Markov random field (GMRF) model. Data are shown after 1985 because few sequences were available before that year. No plots for the C/Aichi lineage and the C/Taylor lineage are presented because of the paucity of sequence data.

We then compared genetic diversity of the lineages of the influenza C virus. Genetic diversity of the C/Kanagawa lineage peaked around 2002 (Figure 2). There was a large outbreak of the influenza C virus between 1999 and 2004 in Japan [53,54]. The great genetic diversity during that time probably reflects the population size during the outbreak. Interestingly, C/Kanagawa lineage strains circulating at the time can be classified into at least three antigenically close, but distinct, groups defined by monoclonal antibodies (Table 1 and Table 2). The variation of antigenicity can be attributed to a large outbreak and great genetic diversity. C/Kanagawa lineage strains collected in or after 2012 were also analyzed for their antigenic characteristics; we found that all strains belonged to a single antigenic group (Table 2). The results are compatible with our finding that genetic diversity has decreased since the peak around 2002 (Figure 2). Along with a decline of genetic diversity of the C/Kanagawa lineage, that of the C/Sao Paulo lineage has increased (Figure 2). Overall, genetic diversity for all lineages of influenza C virus has remained high and constant.

### 3.2. Selective Bottleneck

**Figure 3:**
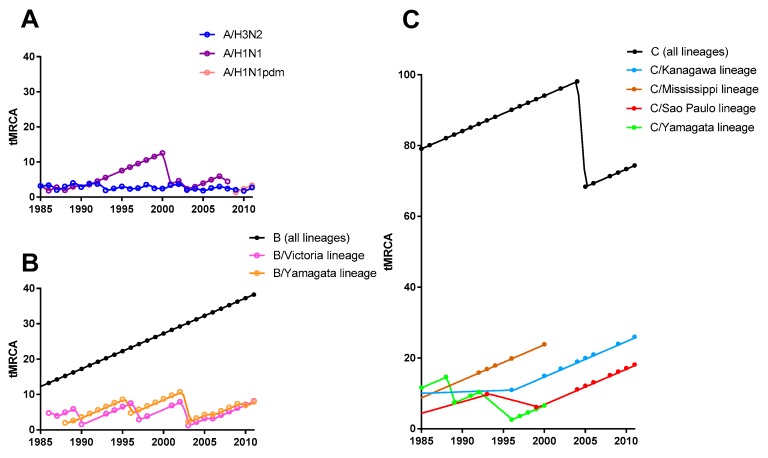
Time to the most recent common ancestor (tMRCA) of influenza A, B, and C viruses. Temporal trend of tMRCA among contemporaneous (same year) and posterior (at least three years) strains for the HA gene of influenza A and B viruses and the HE gene of the influenza C virus, in panel **A**, **B**, and **C** respectively. We did not plot the tMRCA of all influenza A viruses (A/H3N2, A/H1N1, and A/H1N1pdm together) because they are not monophyly. Additionally, tMRCA of influenza A/H1N1 (before 2009) and influenza A/H1N1pdm (in or after 2009) was calculated separately. Data are shown after 1985, since few sequences were available before that year. Since strains collected over four years are required to calculate tMRCA, sequence data up to 2014 yielded tMRCA up to 2011. No plots for the C/Aichi lineage and the C/Taylor lineage are presented because of the paucity of sequence data.

We then compared the time to the most recent common ancestor (tMRCA) of contemporaneous and posterior strains among influenza A, B, and C viruses. tMRCA of all lineages of influenza B and C viruses has been linearly increasing because of co-circulation of multiple lineages (Figure 3B,C). We did not plot the tMRCA of all influenza A viruses (A/H1N1, A/H1N1pdm, and A/H3N2 together) because they are not monophyly; they originated independently from avian or swine influenza viruses. The disappearance of the C/Mississippi lineage and the C/Yamagata lineage in 2005 was responsible for the sudden descent of tMRCA at the time for all lineages of influenza C virus (Figure 3C). In contrast, a short and constant tMRCA of the influenza A virus was observed, which must be due to the continuous emergence of new antigenic variants and the replacement of formerly circulating strains by new strains (Figure 3A). The most fit strains from diversified viruses were selected by the selective bottleneck [47,55]. Short and constant tMRCA of contemporaneous strains, which can be seen for the influenza A virus, reflects a continuous selective bottleneck, while the linear increasing trend of tMRCA of contemporaneous strains means the absence of such selective bottlenecks. Fluctuation of tMRCA for two lineages of the influenza B virus suggests that such a selective bottleneck was present approximately every eight years for each lineage of the influenza B virus (Figure 3B). Similarities and differences between the two lineages of the influenza B virus were discussed well elsewhere [47]. Selective bottleneck was also found at genetic lineage level of the influenza C virus, although tMRCA of each lineage of the influenza C virus has kept increasing, except the C/Yamagata lineage (Figure 3C). The selective bottleneck for the influenza C virus was, therefore, weak and infrequent.

### 3.3. Evolutionary Rate and Selection Pressure

**Figure 4:**
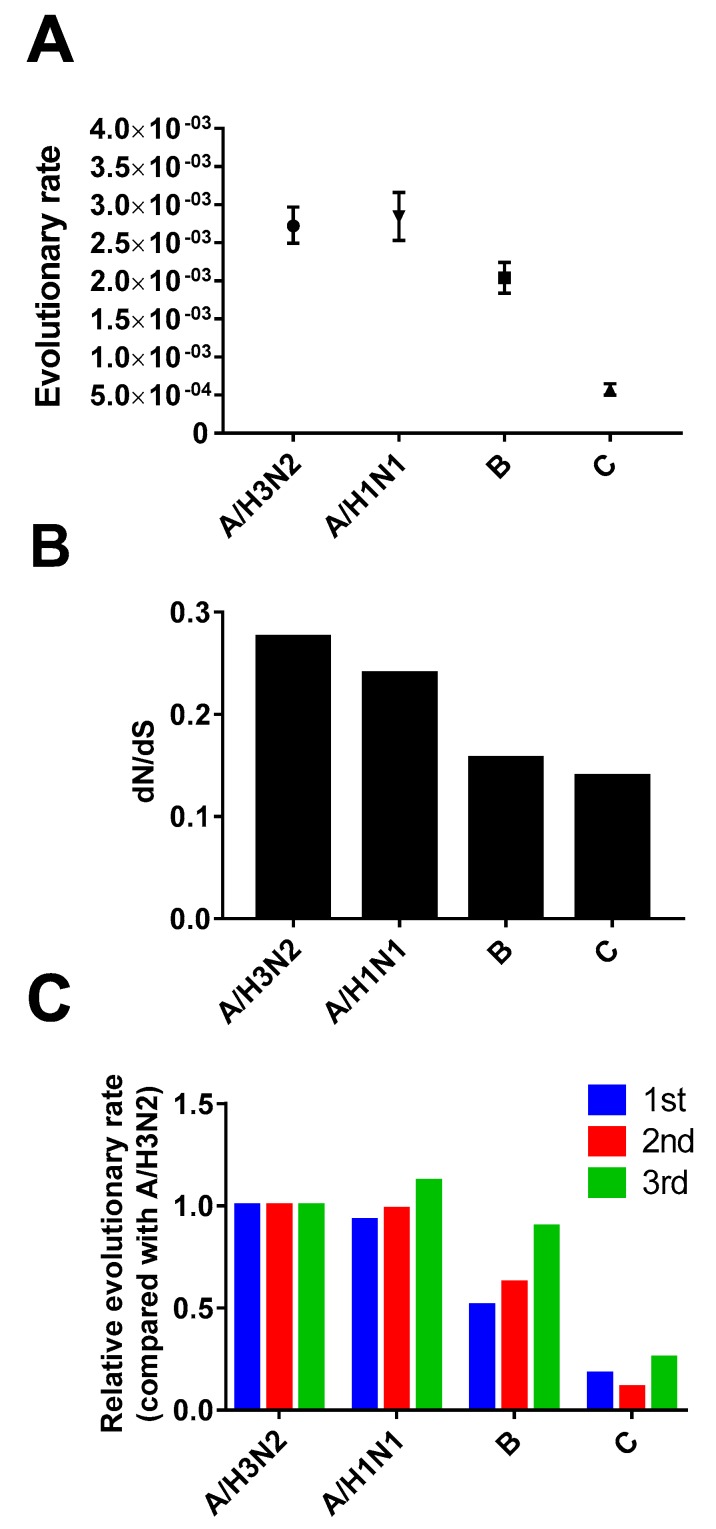
Evolutionary rate and selection pressure of influenza A, B, and C viruses. (**A**) Evolutionary rate (nucleotide substitution per site per year); (**B**) selection pressure (relative rate of non-synonymous and synonymous mutation); and (**C**) relative evolutionary rate of each position in the codon compared with the influenza A/H3N2 virus. In all three panels, results were calculated using data of the HA gene for influenza A and B viruses and the HE gene for the influenza C virus. Error bars show 95% highest posterior density intervals.

We next analyzed and compared evolutionary characteristics of the influenza C virus HE gene with those of the influenza A and B viruses HA gene at the molecular level. The evolutionary rate was the highest for the influenza A virus (HA gene), followed by the influenza B virus (HA gene) and the influenza C virus (HE gene) (Figure 4A). The difference in the evolutionary rate could result from different strengths of selection pressure on the gene. We, therefore, calculated the relative rate of non-synonymous and synonymous mutations (dN/dS) for the viruses. As expected, the selection pressure (dN/dS) was higher for the influenza A virus than for the two other viruses (Figure 4B). The evolutionary rates of the first and second positions in the codon were lower in the influenza B virus than in the influenza A virus, whereas the evolutionary rates of the third position in the codon were similar between the influenza A and B viruses (Figure 4C). Therefore, strong positive selection pressure of the influenza A virus can explain its high evolutionary rate compared with the influenza B virus.

The strength of selection pressure of the influenza C virus (HE gene) was comparable with that of the influenza B virus (HA gene) (Figure 4B). Selection pressure cannot, therefore, explain the slow evolution of the influenza C virus compared with that of the influenza B virus (Figure 4A). It is intriguing that the evolutionary rate of not only the first and second positions in the codon, but also of the third position for the influenza C virus are much lower than the corresponding evolutionary rates in influenza A and B viruses (Figure 4C). The findings indicate that a natural capability of inducing random mutations in the influenza C virus is inferior to the capability of the other two viruses. Possible explanations for this could be a slow replication cycle [56,57], low yield of progeny virus in infected individuals [58], and high fidelity of viral RNA polymerase (no published evidence to our knowledge) of the influenza C virus.

### 3.4. Site-by-Site Selective Pressure on the HE Gene

**Figure 5:**
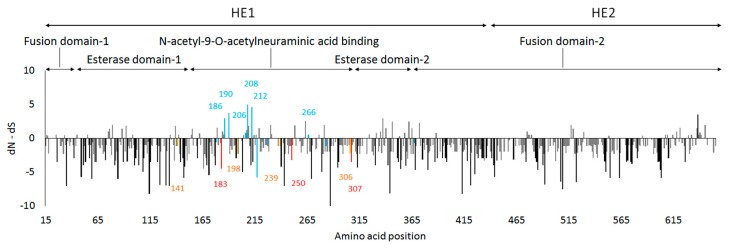
Site-by-site selection pressure for the HE gene of the influenza C virus. Selection pressure (relative number of non-synonymous substitutions minus synonymous substitutions (dN–dS)) on each codon is shown. Amino acid positions between 1 and 14 encoding signal peptides were excluded for the analysis. Selection pressure on antigenic sites (13 positions in total) is colored in blue. Selection pressure on receptor binding sites (11 positions in total) is colored in red (with statistical significance) and orange (no statistical significance). Black and gray bars indicate significant and non-significant selection pressure on the other positions, respectively. Amino acid positions were depicted with bars for antigenic sites under positive selection and receptor binding sites under negative selection.

Site-by-site selective pressure analyses of the HE gene of the influenza C virus found 101 sites under significant negative selection and no sites under significant positive selection (Figure 5). Although there are no sites under significant positive selection, some sites responsible for antigenicity [11] showed positive selection pressure (Supplementary Table S2 and positive blue bars in Figure 5). Additionally, other positions in antigenic sites showed negative selection pressure (Supplementary Table S2 and negative blue bars in Figure 5). Even within antigenic sites, negative selection pressure can be found if there is a strong functional constraint for amino acid substitution [28]. In addition, mutation at a structurally adjacent site may be enough to alter antigenicity [28]. In contrast to antigenic sites, selection pressure for most sites in a receptor-binding domain [59] were under negative selection, as expected (orange and red bars in Figure 5), because mutations in these sites must be fatal for the virus.

We also conducted lineage-by-lineage analyses to see how selection on antigenic sites worked within each lineage (Supplementary Table S2). The analyses still calculated positive values for some antigenic sites, although there was no statistical significance. As mentioned above, there were three antigenically close but distinct groups in the C/Kanagawa lineage around 2002. Amino acids at positions 190 and 212 are associated with the antigenic characteristics (Table 1). We found positive values of dN–dS for the two sites in the C/Kanagawa lineage (Supplementary Table S2). The site could be under weak positive selection by immune pressure.

Finally, we looked at whether the number of *N*-glycosylation sequons (Asn-Xaa-Ser/Thr, where Xaa is any amino acid except Pro) has increased in the HE1 region through evolution to alter antigenicity, as observed in influenza A viruses [30,31]. However, the numbers did not increase at all for any lineages of influenza C virus (Supplementary Figure S1). The potential *N*-linked glycosylation sites has been maintained at six for most influenza C viruses.

## 4. Discussion

In this study, we analyzed the evolutionary pathway of the influenza C virus using 218 viral sequences collected over 68 years. Slow evolution of the influenza C virus is characterized by weak and infrequent selective bottlenecks and a small number of mutations that possibly alter antigenicity. Most sequence data of the influenza C virus are from one country, Japan; while our datasets for influenza A and B viruses consist of sequence data from various parts of the world. There may be selection bias for the influenza C virus. Data collection from a restricted area, however, could lead to overestimation of the strength of selective bottlenecks. Even with the possibility of not underestimation, but overestimation, our analyses found that selective bottlenecks for the influenza C virus are weak and infrequent through its evolution pathway.

The unique evolutionary characteristics of the influenza C virus, have resulted in multiple lineages of the virus co-circulating in different countries. This is similar to the behavior of the influenza B virus whereby multiple lineages of the virus co-circulate, and antigenic variants appear to be slower than in influenza A viruses [33]. In addition, our findings showed a much slower rate of evolution, less frequent selective bottlenecks, and weaker positive selection for the influenza C virus than the influenza B virus.

It is still unknown why the C/Taylor lineage, the C/Aichi lineage, the C/Mississippi lineage, and the C/Yamagata lineage disappeared, whereas the C/Kanagawa lineage and the C/Sao Paulo lineage persist. The existing two lineages experienced surge of genetic diversity (Figure 2). As genetic diversity is associated with effective population size, occasional outbreak with a large number of infected people, which can lead to increase of genetic diversity and fitness of the virus, might be required to prevent it from extinction. It would be intriguing to see whether the high and constant genetic diversity of the influenza C virus would lead to the emergence of novel lineages that would co-circulate with existing lineages.

One limitation of this study is that we analyzed only one segment, the HE gene, of the influenza C virus. Analysis of the only one segment might lead to some loss of information. However, it is difficult to analyze evolutionary characteristics of the virus with the whole genome because of a complicated history of reassortment [18] and an insufficient number of strains with whole-genome sequences. Published studies documented population dynamics of influenza viruses well by analysis with only one segment (the HA gene) for influenza A and B viruses [47,55].

Further questions arise, such as what mechanisms underlie the evolutionary differences between influenza A, B, and C viruses. Selective bottlenecks of the HA/HE gene of influenza viruses at the population level must be mainly driven by the immune pressure of herd immunity [21,55]. Multiple factors must be important to limit the genetic diversity of influenza viruses, including infection incidence rate, seroprevalence, protective immune response, duration of the immunity, and broadness of immunity against heterologous viruses [60,61,62]. A rare selective bottleneck for the influenza C virus suggests unique characteristics of the virus and its infections. Seroprevalence and incidence of the influenza C virus do not seem as high as those of the influenza A virus [5,63,64,65,66]. Further studies are necessary to understand the epidemiological and immunological aspects of influenza C viruses, such as whether multiple exposures throughout life are common, whether immunity against the virus can prevent reinfection, how long the immunity lasts, and whether the infection can induce strain non-specific immunity to constrain genetic diversity. We have limited knowledge of the epidemiological and immunological aspects of influenza C viruses, which must be responsible for its evolutionary characteristics.

Transmission dynamics, such as age at infection and global circulation patterns have also been suggested to affect the evolutionary pathway of influenza A and B viruses [47,67]. There could be unique transmission dynamics of the influenza C virus affecting its evolution in addition to unique virological, immunological, and epidemiological characteristics of the virus. Further studies, combining experimental, epidemiological, and theoretical analyses, are needed for better understanding of the evolutionary and ecological aspects of the influenza C virus.

## Figures and Tables

**Table 1:** Antigenic groups of the C/Kanagawa lineage.

Antigenic Group	Reference Virus	Amino Acid at Position 190	Amino Acid at Position 212	HI Titer of Monoclonal Antibodies							
				J14	J9	Q5	U4	U9	U1	U2	MS22
KA	C/Kanagawa/1/76	D	E	256,000	<20	1280	20	80	<20	<20	<20
AO	C/Aomori/74	D	K	1,024,000	<20	640	<20	40	3200	640	<20
MI	C/Miyagi/77	N	E	1,024,000	<20	6400	2560	64,000	<20	<20	<20

Antigenic characteristics of antigenic groups of the C/Kanagawa lineage are shown by hemagglutinin inhibition (HI) titers of monoclonal antibodies.

**Table 2:** Number of isolates for the C/Kanagawa lineage by antigenic group.

	KA	AO	MI	Undetermined	Total
1996	1	0	0	0	**1**
2000	6	0	0	0	**6**
2001	2	0	0	0	**2**
2002	31	17	3	1	**52**
2003	0	0	0	0	**0**
2004	59	10	9	0	**78**
2005	0	7	0	0	**7**
2006	0	3	2	0	**5**
2007	0	0	0	0	**0**
2008	0	0	0	0	**0**
2009	0	0	0	0	**0**
2010	0	0	0	0	**0**
2011	0	0	0	0	**0**
2012	8	0	0	0	**8**
2013	0	0	0	0	**0**
2014	21	0	0	0	**21**
**Total**	**128**	**37**	**14**	**1**	**180**

Number of isolates and their antigenic groups described in Table 1 are shown. All isolates are from Japan. KA, C/Kanagawa/1/76-like; AO, C/Aomori/74-like; MI, C/Miyagi/77-like.

## Supplementary Materials

The following are available online at www.mdpi.com/1999-4915/8/12/321/s1, Table S1: number of sequences and isolation year by country, Table S2: selection pressure (dN-dS) of antigenic sites, Figure S1: number of *N*-glycosylation sequons in the HE1 region, Data S1: sequence data of influenza C viruses used in this study, Data S2: sequence data of influenza A/H3N2 viruses used in this study, Data S3: sequence data of influenza A/H1N1 viruses used in this study, Data S4: sequence data of influenza B viruses used in this study.
