# Supplementary material for: Analyses of Evolutionary Characteristics of the Hemagglutinin-Esterase Gene of Influenza C Virus during a Period of 68 Years Reveals Evolutionary Patterns Different from Influenza A and B Viruses
Source: Viruses. 2016 Nov 26;8(12):321. doi: 10.3390/v8120321 (PMC5192382; doi:10.3390/v8120321)
Supplement: Supplementary file 1 [file viruses-08-00321-s001.zip › viruses-148034-supplementary-final/viruses-148034-supplementary-final.docx]

Supplementary Materials: Analyses of Evolutionary Characteristics of the Hemagglutinin-Esterase Gene of Influenza C Virus during a Period of 68 Years Reveals Evolutionary Patterns Different from
Influenza A and B Viruses

Yuki Furuse, Yoko Matsuzaki, Hidekazu Nishimura and Hitoshi Oshitani

**Table S1.** Number of sequences and isolation year by country.

| **Country** | **Number of Sequences** | **Year** |
| --- | --- | --- |
| Australia | 32 | 2008–2014 |
| Brazil | 1 | 1982 |
| China | 1 | 1981 |
| Fiji | 1 | 2012 |
| France | 1 | 1967 |
| Greece | 1 | 1979 |
| India | 1 | 2011 |
| Japan | 137 | 1964–2014 |
| Philippines | 8 | 2011–2013 |
| Singapore | 4 | 2006 |
| South Africa | 4 | 1966 |
| Spain | 10 | 2009 |
| UK | 2 | 1983 |
| USA | 15 | 1947–2012 |
| **Total** | **218** | **1947–2014** |

**Table S2.** Selection pressure (dN–dS) of antigenic sites.

| **Antigenic Site** | **Amino Acid Position** | **dN–dS (*p*-value)** | | | | | | |
| --- | --- | --- | --- | --- | --- | --- | --- | --- |
|  |  | **All Lineages** | **C/Aichi Lineage** | **C/Kanagawa Lineage** | **C/Mississippi Lineage** | **C/Sao Paulo Lineage** | **C/Taylor Lineage** | **C/Yamagata Lineage** |
| A-1 | 186 | 2.94 (0.15) | 0 (−) | 2.04 (0.20) | 0 (−) | 0 (−) | 0 (−) | 0 (−) |
|  | 187 | 0 (−) | 0 (−) | 0 (−) | 0 (−) | 0 (−) | 0 (−) | 0 (−) |
|  | 190 | 3.72 (0.070) | 0 (−) | 3.75 (0.065) | 0 (−) | 0 (−) | 0 (−) | 0 (−) |
|  | 206 | 0.79 (0.50) | 0 (−) | −0.41 (0.60) | 0 (−) | 0 (−) | 0 (−) | 0.78 (0.46) |
|  | 245 | 0 (−) | 0 (−) | 0 (−) | 0 (−) | 0 (−) | 0 (−) | 0 (−) |
|  | 283 | −1.18 (0.28) | 0 (−) | −1.15 (0.30) | −1.17 (0.29) | −1.13 (0.30) | 0 (−) | 0 (−) |
| A-2 | 367 | −0.71 (0.49) | 0 (−) | 0.47 (0.71) | −1.18 (0.28) | 0 (−) | 0 (−) | 0 (−) |
| A-3 | 178 | −0.59 (0.60) | 0 (−) | −0.58 (0.61) | 0 (−) | 0 (−) | 0 (−) | 0 (−) |
|  | 208 | 4.97 (0.057) | 0 (−) | 0.47 (0.29) | 0 (−) | 0.95 (0.50) | 0 (−) | 0 (−) |
|  | 212 | 4.60 (0.073) | 0.48 (0.70) | 2.89 (0.12) | 0 (−) | −1.13 (0.30) | 0 (−) | 1.41 (0.39) |
|  | 217 | −5.83 (0.010) | 0 (−) | −0.51 (0.55) | 0 (−) | 0 (−) | 0 (−) | −3.80 (0.018) |
| A-4 | 226 | −1.00 (0.33) | 0 (−) | −1.00 (0.33) | 0 (−) | 0 (−) | 0 (−) | 0 (−) |
|  | 266 | 0.50 (0.59) | −0.50 (0.55) | 0 (−) | 0.50 (0.67) | 0 (−) | 0 (−) | 0.50 (0.67) |

Sites where dN–dS showed positive and negative values were inferred as positively and negatively selected, respectively. When no mutation was observed, dN–dS was 0 and *p*-value could not be calculated.

**Figure S1.** Number of *N*-glycosylation sequons in the hemagglutinin-esterase 1 (HE1) region, showing the average number of *N*-glycosylation sequons in the HE1 region for each lineage over a window of 10 years.
